# Development of a multicomponent intervention program to prevent delirium in intensive care unit patients

**DOI:** 10.1186/2197-425X-3-S1-A714

**Published:** 2015-10-01

**Authors:** A Wassenaar, M van den Boogaard, P Rood, L Schoonhoven, P Pickkers, UNDERPIN-ICU Study Group

**Affiliations:** Radboud University Medical Center, Intensive Care Medicine, Nijmegen, Netherlands; University of Southampton, Faculty of Health Sciences, Southampton, United Kingdom; Radboud University Medical Center, Scientific Institute for Quality of Healthcare, Nijmegen, Netherlands

## Introduction

Delirium is common in Intensive Care Unit (ICU) patients and associated with poor outcome. Therefore delirium prevention is imperative. Interventions targeting several delirium risk factors represent a promising strategy for prevention. In preparation for a stepped wedge randomized controlled trial to study the effect of a multicomponent intervention program on ICU delirium, we developed an intervention program which is aimed at delirium prevention to ultimately increase the number of delirium free days.

## Methods

A concept intervention program targeting cognitive impairment, sleep deprivation, immobility, and visual and hearing impairment was developed based on a review. As most of the founded studies were conducted in non-ICU patients, we studied experts' opinion about the feasibility and completeness of the concept program in ICU patients during a modified RAND/UCLA Appropriateness Method Delphi study [1]. This incorporated a 2 round anonymous expert panel to reach consensus about the intervention program. A disagreement index (DI) < 1 indicates agreement. To determine the practicability and burdening of cognitive training, an element of the interventions targeting cognitive impairment, a prospective cohort pilot study was performed. All 11 cognitive trainings were tested for practicability and burdening 4 times: 2 times in delirious and 2 times in non-delirious patients. Patients were screened for delirium with the validated confusion assessment method-ICU. Practicability and burdening were determined using 5-point Likert scale, and open questions, vital signs, and time investment.

## Results

The Delphi study comprised 38 experts (Table). There was 100% completion of the questionnaires in round 1 and 79% in round 2. The mean overall DI was 0.35 ± 0.25. The expert group agreed on the feasibility of the interventions targeting sleep deprivation, immobility, and visual and hearing impairment, but was uncertain about cognitive training which targets cognitive impairment. Preliminary, in total 44 ICU patients (mean age 69 ± 9.6) were included in the pilot study of whom 55% were medical - and 45% surgical patients, and 50% with delirium. Overall, patients rated the cognitive training as practicable and not burdening. In the next round we will study the practicability and burdening for ICU nurses of providing cognitive training, and finally decrease the number of trainings based on the results.

## Conclusions

A feasible multi component intervention program to prevent ICU delirium was developed based on expert consensus and a pilot study.

**Table 1:** Participants characteristics Delphi study.

Characteristics	Delphi round 1	Delphi round 2
Age, years (SD)	42 ± 7.7	41 ± 7.7
Male, N (%)	18 (47.4)	13 (43.3)
Education, N (%) [A] Secondary vocational education; [B] Higher professional education; [C] Academic level	[A] 0; [B] 23 (60.5); [C] 15 (39.5)	[A] 0; [B] 19 (63.3); [C] 11 (36.7)
Profession, N (%) [A] ICU nurse; [B] Physical therapist; [C] Physician; [D] Delirium researcher	[A] 12 (31.6); [B] 12 (31.6); [C] 12 (31.6); [D] 2 (5.2)	[A] 9 (30); [B] 11 (36.7); [C] 9 (30); [D] 1 (3.3)
Disagreement index (SD)	0.35 ± 0.25	0.31 ± 0.20
